# How to manage aspergillosis in non-neutropenic intensive care unit patients

**DOI:** 10.1186/s13054-014-0458-4

**Published:** 2014-07-25

**Authors:** Matteo Bassetti, Elda Righi, Gennaro De Pascale, Raffaele De Gaudio, Antonino Giarratano, Tereesita Mazzei, Giulia Morace, Nicola Petrosillo, Stefania Stefani, Massimo Antonelli

**Affiliations:** Infectious Diseases Division, Santa Maria Misericordia University Hospital, 33100 Udine, Italy; Istituto di Anestesiologia e Rianimazione, Università Cattolica-Policlinico Universitario A.Gemelli, 00100 Roma, Italy; Department of Health Sciences, Anesthesiology and Intensive Care Section, University of Florence, 50100 Firenze, Italy; Anesthesia, Analgesia and Intensive Care Division, P.Giaccone University Hospital, School of Medicine DiBiMef-University of Palermo, 90100 Palermo, Italy; Department of Health Sciences - Section of Clinical Pharmacology and Oncology, University of Florence, 50100 Firenze, Italy; Department of Health Sciences, Università degli Studi di Milano, 20100 Milano, Italy; Second Division, Lazzaro Spallanzani National Institute for Infectious Diseases, 00100 Roma, Italy; Department of Bio-medical Sciences, University of Catania, 95100 Catania, Italy

## Abstract

Invasive aspergillosis has been mainly reported among immunocompromised patients during prolonged periods of neutropenia. Recently, however, non-neutropenic patients in the ICU population have shown an increasing risk profile for aspergillosis. Associations with chronic obstructive pulmonary disease and corticosteroid therapy have been frequently documented in this cohort. Difficulties in achieving a timely diagnosis of aspergillosis in non-neutropenic patients is related to the non-specificity of symptoms and to lower yields with microbiological tests compared to neutropenic patients. Since high mortality rates are typical of invasive aspergillosis in critically ill patients, a high level of suspicion and prompt initiation of adequate antifungal treatment are mandatory. Epidemiology, risk factors, diagnostic algorithms, and different approaches in antifungal therapy for invasive aspergillosis in non-neutropenic patients are reviewed.

## Review

### Introduction

Invasive aspergillosis (IA) is an opportunistic infection that occurs mainly among patients with hematological malignancies, most notably during prolonged periods of neutropenia, but also in subjects with solid tumors, critical illness, and HIV/AIDS, and those undergoing allogeneic stem cell transplantation and solid-organ transplantation [1,2]. In recent years, however, IA has increasingly been recognized as an emerging disease of non-neutropenic patients and in patients admitted to the ICU, even in the absence of an apparent predisposing immunodeficiency [3-8]. Although not uncommon, the features of IA among immunocompetent patients differ greatly from those of IA in neutropenic patients. The epidemiology, clinical characteristics, outcomes, and prognosis are not well known in immunocompetent patients. In the ICU, the incidence of IA ranges from 0.3% to 5.8% [4,5] with an overall mortality rate exceeding 80% [9].

Several recent case series and single-center cohort reports have documented the expansion of patient populations at risk for IA that are different from the traditionally recognized risk groups. They include patients with chronic obstructive pulmonary disease (COPD) and other chronic lung or connective tissue diseases requiring corticosteroid therapy, decompensated liver cirrhosis, and solid cancer with or without treatment [10,11].

The diagnosis of IA in non-neutropenic critically ill patients is difficult because signs and symptoms are non-specific, and the initiation of additional diagnostic examinations is often delayed because of a low clinical suspicion. A high level of suspicion is needed to obtain an early diagnosis and a timely therapeutic intervention. A better understanding of the population at risk and the spectrum of diseases caused by IA in non-neutropenic patients may help to improve the outcome of this potentially treatable disease.

In this review, we describe the epidemiology of and the risk factors for pulmonary IA in non-neutropenic patients, limitations and advances in the diagnostic process, and the different approaches in antifungal therapy, including the main pharmacological properties of different antifungal drugs.

### Epidemiology

Despite a documented increase in the incidence of IA in ICUs, different rates are reported among subsets of ICU patients. Indeed, a high prevalence (17%) of IA has been observed in a cohort of 67 patients with severe hospital-acquired pneumonia admitted to the ICU [12]. Among 40 critically ill patients with confirmed H1N1 infection, 9 (23%) developed IA 3 days after ICU admission [13].

Retrospective, autopsy-controlled studies showed interesting results. Roosen and colleagues [14] studied causes of death in the ICU, revealing 15 cases of IA, 5 of which were undiagnosed before death, among 100 autopsies. In a retrospective study, 127 patients out of 1,850 admissions (6.9%) had microbiological or histopathological evidence of *Aspergillus* during their ICU stay [5]. Postmortem examination was done in 47 out of 71 patients, and 27 (59%) were identified with IA.

In a study comparing neutropenic and non-neutropenic patients with an IA diagnosis during a 6-year period, Cornillet and colleagues [6] found a mean number of 15 IA cases per year; of these, approximately half were in the ICU. In an Italian study conducted in two mixed ICUs during 2 years, the incidence of IA was 0.2%, much lower than in other reports from similar ICUs [15].

Risk factors for IA in non-neutropenic patients admitted to the ICU include prolonged treatment with corticosteroids before admission, COPD, liver cirrhosis with prolonged ICU stay (>7 days), solid organ cancer, HIV infection, and lung transplantation [16]. However, most of these factors are frequent among non-neutropenic critically ill patients. An intriguing hypothesis on the cause of immunosuppression in the apparently immunocompetent patient with multiple-organ dysfunction relates to the biphasic response to sepsis. Indeed, the initial hyperinflammatory phase is followed by relative immunoparalysis. This latter process is characterized by neutrophil deactivation, and it may put the patient at risk of developing opportunistic infections, such as IA [17].

### Risk factors

One of the most important risk factors for IA in non-neutropenic patients is COPD [7]. Patients with COPD are susceptible to *Aspergillus* colonization of the lower tract of the respiratory airway and under particular circumstances this may lead to invasive infection [18]. COPD patients present alterations in lung structure, an impaired immunologic response, reduced mucociliary clearance and mucosal lesions. Moreover, they are prone to frequent hospitalization, broad-spectrum antibiotic treatment and invasive procedures. All these factors could explain the high incidence of aspergillosis in COPD [7]. Of note, they are frequently treated with corticosteroids and both inhaled and systemic therapy have been described as another important risk factor for aspergillosis [19,20]. Steroids are able to accelerate the *in vitro* growth of *Aspergillus* spp. since both the innate and acquired immune responses are impaired [21]. Vandewoude and colleagues [22] defined a total daily dose ≥20 mg prednisone or equivalent among criteria for defining cases of IA. Both compensated and decompensated cirrhosis have been described as risk factors for IA and impaired phagocytosis has been proposed as a possible explanation in these groups [23,24]. Diabetes has been observed as another risk factor [22]. Impaired innate and acquired immunity caused by hyperglycemia may explain this observation [25]. Several authors report alcoholism and malnutrition as other possible risk factors for IA [22,26].

Patients in the ICU are subjected to several therapies (for example, broad spectrum antibiotics, mechanical ventilation) and/or maneuvers (for example, insertion of central venous catheter), which may affect the immune system defenses. Even though some of these conditions have been described as possible contributors, additional factors may be required for the development of IA [5,16,26].

Immunosuppression has been described as a late stage of the biphasic response to sepsis and multiple organ failure syndrome [27]. Hartemink and colleagues [17] first proposed the association between this condition and IA development. This could be one of the main reasons why aspergillosis is frequent among patients not considered immunocompromised by classic criteria.

### Clinical diagnosis and case definition

Clinical manifestations of IA (for example, fever, cough, purulent sputum) may be initially indistinguishable from those of bacterial bronchopneumonia [28]. The recovery of the same *Aspergillus* species from several respiratory samples in the course of antibiotic-resistant pneumonia in patients with risk factors is clearly evocative of the diagnosis [10]. Therefore, it has been proposed that the isolation of an *Aspergillus* species from the respiratory tract in critically ill patients with risk factors (COPD after corticosteroid exposure, severe underlying disease) and clinical features of pneumonia should indicate a probable IA case.

The presence of a persistent pulmonary infection despite broad-spectrum antibiotics or abnormal thoracic imaging by CT scanning together with one of the risk factors should trigger further diagnostic exploration through collection of respiratory secretions and/or laboratory markers. Invasive infections in patients with negative cultures might be supported by positive molecular and serological tests, such as *Aspergillus* PCR and galactomannan (GM) antigen, which requires at least two sequentially positive samples. Radiological findings can be non-specific in non-neutropenic patients, and of the typical imaging findings observed in neutropenic patients, the air crescent sign was seen in only a small proportion of cases, while the halo sign was very rarely observed. The halo sign and air crescent sign in thoracic CT scans have a high sensitivity (80%) and specificity (60 to 98%) for IA among neutropenic patients with pulmonary infection [29]. In non-neutropenic patients, a lower sensitivity (5 to 24%) is reported in the literature and these signs are less frequently observed [30,31]. Bronchoscopy manifestations were also non-specific in non-neutropenic patients, with a lack of consistent endoscopic features [31].

The diagnosis of IA is particularly problematic. According to the revised definitions for invasive fungal disease of the European Organization for Research and Treatment of Cancer/Invasive Fungal Infections Cooperative Group and the National Institute of Allergy and Infectious Diseases Mycoses Study Group (EORTC/MSG) Consensus Group, IA is categorized into proven, probable, and possible invasive fungal disease [32]. These diagnostic criteria have proven to be useful in research and practice in severely immunocompromised patients. The lack of specific criteria for diagnosing IA in non-neutropenic patients, however, hampers the timely initiation of appropriate antifungal therapy and may, as such, compromise the odds of survival. Recently, Blot and colleagues [33] externally validated a clinical diagnostic algorithm (Table 1) that aims to discriminate colonization from probable IA in ICU patients with *Aspergillus*-positive endotracheal aspirate cultures.

**Table 1:** **Clinical algorithm for the diagnosis of invasive aspergillosis in non-neutropenic patients**

**Proven invasive pulmonary aspergillosis**	
**●**	**Follow EORTC/MSG criteria**
**Putative invasive pulmonary aspergillosis (all four criteria must be met)**	
**●**	**1. Aspergillus-positive lower respiratory tract specimen culture**
**●**	**2. Compatible signs and symptoms (one of the following)**
	**Fever refractory to at least 3 days of appropriate antibiotic therapy**
	**Recrudescent fever after a period of defervescence of at least 48 hours** **while still on antibiotics and without other apparent cause**
	**Pleuritic chest pain**
	**Pleuritic rub**
	**Dyspnea**
	**Hemoptysis**
	**Worsening respiratory insufficiency in spite of appropriate antibiotic therapy and ventilatory support**
**●**	**3. Abnormal medical imaging by portable chest X-ray or CT scan of the lungs**
**●**	**4. Either 4a or 4b**
	**4a. Host risk factors (one of the following conditions)**
	**Neutropenia preceding or at the time of ICU admission**
	**Underlying hematological or oncological malignancy treated with cytotoxic agents**
	**Glucocorticoid treatment (prednisone equivalent, 20 mg/day)**
	**Congenital or acquired immunodeficiency**
	**COPD, decompensated cirrhosis**
	**4b. ** **Semiquantitative ** ***Aspergillus*** **-positive culture of BAL fluid without bacterial growth together with a positive cytological smear showing branching hyphae**
***Aspergillus*** **respiratory tract colonization**	
**●**	**When >1 criterion necessary for a diagnosis of putative IPA is not met, the case is classified as** ***Aspergillus*** **colonization**

BAL, bronchoalveolar lavage; COPD, chronic obstructive pulmonary disease; CT, computed tomography; EORTC/MSG, European Organization for Research and Treatment of Cancer/Invasive Fungal Infections Cooperative Group and the National Institute of Allergy and Infectious Diseases Mycoses Study Group; IPA, invasive pulmonary aspergillosis.

### Microbiological diagnosis

The microbiological diagnosis of aspergillosis can be achieved using conventional and molecular approaches, including antigen detection and PCR assays [34,35]. The direct examination of clinical specimens by microscopy is particularly relevant to observe the fungal parasitism; this morphology can allow a presumptive diagnosis of aspergillosis. Microscopy is generally performed using wet preparations (potassium hydroxide, calcofluor) and Wright or Giemsa stains. Other specialized stains, like periodic acid-Schiff or Gomori methenamine silver, are generally performed in the histology laboratory [34].

Since *Aspergillus* microscopic fungal elements can be confused with those of *Fusarium* and *Scedosporium* species, conventional culture methods are essential for isolating and identifying the etiological agent. Identification is largely based on an accurate analysis of the macro- and microscopic features of the colonies: the size, color and shape of the colony, microscopic visualization of conidiophores and conidial heads, morphology size and color of the conidia are important features useful to identify the isolate at the species level [34,36]. More recently, DNA sequencing and the matrix-assisted laser desorption/ionization-time of flight mass spectrometry proteomic approach have proven to be useful tools to identify non-sporulating isolates or isolates with atypical morphology [37,38]. It should be remembered that a negative result by both microscopy and culture does not exclude an active infection. The availability of clinical *Aspergillus* spp. isolates allows *in vitro* antifungal susceptibility testing, which can be useful to detect the emergence of resistance, especially to triazoles [39].

The detection of antibodies against *Aspergillus* is strongly dependent on the immune status of the patient and has been proven to be of little value in the diagnosis of IA [35].

The Platelia *Aspergillus* enzyme immunoassay (Bio-Rad Laboratories, Redmond, WA, USA) reveals the presence of GM, a polysaccharide of the outer cell wall layer of *Aspergillus*, in patients with suspected aspergillosis [34,35]. Because GM is produced at the apical hyphae of actively growing *Aspergillus*, the performance of this immunoassay decreases when antifungal therapy is successful [34]. GM can be detected in body fluids, but serum levels in non-neutropenic patients do not seem to be accurate because circulating neutrophils are able to clear the antigen. Meersseman and colleagues [40] demonstrated a high sensitivity and specificity of GM in bronchoalveolar lavage (BAL) for the diagnosis of IA; the sensitivity of BAL GM was 88% compared with 40% for serum GM. GM detection in BAL is, therefore, a valuable tool for the diagnosis of IA also in non-neutropenic patients. Alternatively, we could test for 1,3-β-D-glucan, a cell-wall component of many fungi, in sera of patients with suspected aspergillosis.

Encouraging results have been obtained using PCR techniques (that is, real time, nested) to detect *Aspergillus* DNA in the sera of patients with proven and probable aspergillosis. Although these tests have the advantage of being non-invasive and EU approved real time PCR kits could overcome the problems related to the absence of a standardized methodology, molecular detection of nucleic acids is not yet considered sufficiently reliable for use in the diagnosis of IA [32,41]. Moreover, conflicting results have been described in cases of histologically proven invasive aspergillosis when the PCR method was performed on BAL [42,43].

### Therapeutic approaches

Prompt administration of appropriate antifungal therapies for IA are immensely important to limit its mortality rate, which ranges from 60% to 90% [16]. Hence, even patients without classic risk factors (that is, COPD, steroids and immunosuppressive agent use, hepatic failure, ICU-related immunoparalysis) should start adequate antifungal therapy upon suspicion of IA before obtaining definitive proof of infection. Early treatment initiation according to first-line therapy, at the stage of possible infection, has been reported to be associated with improved outcome in a retrospective cohort of 289 IA cases characterized by different predictors of death [44].

Additionally, with the exclusion of neutropenic and allogenic hematopoietic stem cell transplantation recipients, the usefulness of anti-fungal prophylaxis has not been established. In non-neutropenic critically ill patients admitted to the ICU, this preventive approach is thus not recommended [45].

Unlike the setting of febrile neutropenic episodes, there is no consensus about the exact time frame to use before starting empirical therapy without any diagnostic support in other critically ill patients at risk of IA [46]. In a 6-year French survey, non-neutropenic patients with IA were less likely to show symptoms; nevertheless, microbiological samples, antigenemia assays and thoracic CT findings had sensitivities similar to those of neutropenic patients [6]. In non-neutropenic patients, therefore, a pre-emptive approach based on microbiological biomarkers (GM, *Aspergillus* PCR, 1,3-beta-glucan) may be useful and should be implemented for early detection and prompt treatment of invasive fungal infections in the ICU [11,47].

Three classes of antifungal agents are available for the treatment of IA: azoles (voriconazole, posaconazole, itraconazole), amphotericin B, and echinocandins (Table 2). Current guidelines recommend voriconazole as first-line treatment for IA, including severely critically ill patients, where intravenous administration is preferred [48]. During the past 10 years, voriconazole use has been widely and progressively used. In a randomized controlled trial in 2002 involving 277 patients with IA mainly affected by hematologic diseases, voriconazole use compared with amphotericin B was associated with statistically significant higher successful outcomes, survival rates and fewer severe adverse events [49]. Voriconazole was the main antifungal used for the treatment of IA during a large prospective surveillance study conducted in North America between 2004 and 2008 [50]. In a retrospective study of 289 IA patients, the authors observed that, after October 2002 (when amphotericin B formulations where replaced by voriconazole as the first-line anti-*Aspergillus* treatment), the overall survival rate increased from 47.5% to 60.4% (*P* = 0.01), without concomitant modifications regarding diagnostic strategy [44]. Recently, Burghi and colleagues [51] analyzed data from 67 patients admitted to ICU with acute respiratory failure due to infection with *Aspergillus* spp*.* Voriconazole therapy was independently associated with lower mortality, confirming its primary role in the management of IA. A large retrospective cohort study investigating risk factors and outcome of ICU patients with IA (excluding those with classic risk factors) showed that a 1-day delay in starting effective antifungal therapy was associated with a longer length of stay (by 1.28 days) and 4% higher total costs per day (*P* < 0.001). Voriconazole was the most frequently prescribed antifungal and its use appeared to improve the abovementioned outcome measures [52]. Data collected from a large multinational randomized controlled trial, involving mainly hematological and transplanted patients, confirmed better outcomes for patients treated with voriconazole compared with conventional amphotericin B, even though total treatment costs were similar [53].

**Table 2:** **Treatment of invasive aspergillosis in non-neutropenic ICU patients** [48]

**Setting**	**First choice**	**Alternatives**
Primary therapy	Voriconazole (6 mg/kg every 12 hours intravenously on day 1, then 4 mg/kg every 12 hours intravenously)	Liposomial amphotericin B (3–5 mg/kg/day intravenously) or Echinocandins (usual dosage)
Salvage therapy	Combination of voriconazole plus amphotericin B/echinocandins	

Itraconazole is considered a second-line therapeutic agent for the treatment of IA, especially in severely ill patients. However, its oral use has been described in non-life-threatening infections where the patients had already been stabilized with a more potent agent [54]. Posaconazole is a broad-spectrum triazole with anti-*Aspergillus* activity similar to that of voriconazole. In a retrospective case–control study involving 193 patients with IA and other mycoses, its use was associated with a 42% survival rate [48]. However, limited clinical experience with it and the absence of intravenous formulations strongly reduce its applicability in critically ill patients. Although rare, triazole resistance in *Aspergillus* spp. (that is, *Aspergillus fumigatus*) has been reported. In these cases, alternative antifungal treatment should be adopted [55].

Before the introduction of voriconazole, amphotericin B was the main treatment for IA. The deoxycholate formulation was associated with severe nephrotoxicity, infusion-related adverse events (fever, chills, arthralgias), and poor outcomes. Three lipid formulations have been approved and are associated with fewer renal toxicity and drug-related side effects, although optimal dosages have not been defined for any of these compounds [56]. In a population of 201 patients with confirmed IA, Cornely and colleagues [57] demonstrated that patients who received a high dose of liposomal amphotericin B (10 mg/kg/day) did not experience higher cure rates compared with standard doses, although relevant nephrotoxicity was observed. In a retrospective cohort of 16 COPD patients with IA treated with a deoxycholate formulation, the mortality rate was 100%, mainly due to septic shock or multiorgan failure. This poor prognosis raised doubts about the need for higher doses or lipid formulations in specific subgroups of patients [3].

All echinocandins have been shown to have *in vitro* and *in vivo* activity against *Aspergillus* spp*.* However, only caspofungin is approved for the treatment of IA in patients who are intolerant to first-line compounds [48]. In two phase II studies involving leukemic and hematopoietic stem cell transplantation patients treated with caspofungin, 12-week survival exceeded 50% [58]. Although still not approved, two other echinocandins (anidulafungin and micafungin) are used in clinical practice, especially with non-neutropenic patients. In breakthrough IA and refractory diseases, combination therapy (for example, echinocandin plus voriconazole or liposomal amphotericin B) may be considered.

Although limited by the use of historical controls, some studies suggest the benefits of voriconazole-caspofungin combinations [59,60]. Furthermore, in a subgroup of 40 solid organ transplant recipients, this combination, as first-line therapy, was associated with significantly reduced mortality compared with amphotericin B [61]. Similarly, a caspofungin-amphotericin B combination has been used with a more than 50% favorable antifungal response [62,63]. On the other hand, no clinical data support triazole-amphotericin B combinations due to possible antagonistic interactions. A phase III clinical trial investigating the effectiveness of a voriconazole-anidulafungin combination did not provide conclusive results [64]. All-cause mortality rates at week 6 for proven or probable IA cases was 19.3% in the voriconazole-anidulafungin group versus 27.5% in the voriconazole group. A recent meta-analysis on combination therapy for IA concluded that the available clinical evidence is not conclusive and of moderate strength [65].

The optimal duration of IA treatment is not known. Early assessment of treatment response is essential to confirm effectiveness. The site of infection, immunosuppressive status, baseline clinical conditions and subsequent therapeutic interventions may all influence physicians’ decisions. Generally, antifungals are not interrupted until all clinical signs have disappeared and radiological abnormalities have stabilized.

Recommendations regarding management of IA in non-neutropenic patients principally derive from evidence from hematological population studies. Large observational cohort studies and interventional trials are needed in order to define the most appropriate therapeutic approaches in non-neutropenic critically ill ICU patients.

#### Pharmacological properties of voriconazole

One of the main pharmacokinetic parameters of voriconazole is its excellent oral bioavailability [66-68]. It possesses the highest bioavailability among triazoles (>85 to 90%), which results in rapidly high plasma concentrations. The absorption of voriconazole is not affected by gastric pH but is decreased by co-administration with food [69]. Peak plasma concentrations close to steady state are rapidly achieved via an intravenous loading dose followed by a maintenance dose within the first 24 hours of administration, but only after 5 to 7 days following multiple oral administrations. Thus, the intravenous route seems to be preferable for initial administration of voriconazole in critically ill patients suffering from IA in order to achieve therapeutic voriconazole levels as early as possible.

An analysis of pharmacokinetic data from several voriconazole clinical trials showed that median voriconazole plasma concentrations in older patients (>65 years) were approximately 80% to 90% higher than those in younger patients after both intravenous and oral administration [70]. The estimated voriconazole oral bioavailability was lower (60%) than previously observed, which might be explained by altered gastrointestinal function, which is frequent in onco-hematological patients [70]. Voriconazole is mainly eliminated by the liver, while kidney elimination is negligible, and less than 5% of the active drug is found in urine.

Voriconazole achieves therapeutically effective concentrations in the epithelial lining fluid after standard doses [71-73]. A recent experience assessing trough voriconazole concentrations in plasma and pulmonary epithelial lining fluid of lung transplant recipients receiving oral voriconazole showed a very high mean ± standard deviation epithelial lining fluid/plasma ratio [74]. This may by predictive of its efficacy in the treatment of pulmonary aspergillosis. Additionally, voriconazole is extensively transported across the blood–brain and blood-eye barriers [73,75,76]. A recent reference laboratory experience of clinically achievable voriconazole concentrations within cerebrospinal fluid (CSF) showed that, among 173 samples, the median quantifiable CSF level was 2.47 mg/L [77]. The effective levels in CSF may support the results of a recent retrospective analysis assessing the efficacy of voriconazole in the treatment of 192 fungal central nervous system infections that documented a success rate of 48% [78].

Variability of voriconazole serum concentrations is mainly due to metabolism via the CYP2C19 P450 enzyme [79-81]. Standard dosing in adults is outlined in Table 3.

**Table 3:** **Standard dosing in adults and children**

**Adults and adolescents (>12 years and >50 kg)**	
Loading dose, for the first 24 hours	
●	IV: 6 mg/kg every 12 hours
●	Oral >40 kg: 400 mg every 12 hours
●	Oral <40 kg: 200 mg every 12 hours
Maintenance dose	
●	IV: 4 mg/kg every 12 hours
●	Oral >40 kg: 200 mg every 12 hours
●	Oral <40 kg: 100 mg every 12 hours

IV, intravenous.

If a response to voriconazole is inadequate, the maintenance oral dose may be increased to 300 mg every 12 hours for patients weighing over 40 kg and to 150 mg every 12 hours for those <40 kg. Dose adjustment is required in case of hepatic failure. According to the prescribing information summary, dose adjustments are required for patients with mild to moderate hepatic dysfunction (Child-Pugh class A and B). The standard loading dose should be provided to these patients, but maintenance doses should be reduced by 50%. Studies have not adequately evaluated the safety of voriconazole in severe liver disease (Child-Pugh class C) [82]. Caution should be exercised when administering the intravenous formulation to critically ill patients with renal dysfunction due to the presence of the solubilizing excipient sulfobutylether-beta-cyclodextrin. Indeed, two recent clinical experiences assessing the safety of intravenous voriconazole in patients with compromised renal function showed that the route of administration and baseline renal function were not predictors of worsening renal dysfunction [83,84].

Although voriconazole has many drug interactions, their clinical management can be relatively simple (Table 4) [85].

**Table 4:** **The main drugs interacting with voriconazole**

**Drug**	**Interaction with voriconazole and management strategy**
Drugs contraindicated	
Astemizole, cisapride, ergot alkaloids, quinidine, sirolimus, terfenadine	Their levels are increased by voriconazole, avoid co-administration. Switch to a drug with no or with predictable interactions (for example, cyclosporine)
Carbamazepine, long-acting barbiturates, rifampicin	They decrease voriconazole levels, avoid co-administration. Switch to a drug with no interactions (for example, levetiracetam)
Rifabutin	Co-administration decreases voriconazole levels and increases rifabutin levels (contraindicated according to FDA, not according to EMA, see below), avoid co-administration
Drugs not contraindicated but if co-administered the dose of voriconazole must be modified (increased)	
Phenytoin	Increase voriconazole oral maintenance dose from 200 mg to 400 mg every 12 hours (100–200 mg every 12 hours if <40 kg) and intravenous maintenance dose to 5 mg/kg every 12 hours; monitor for phenytoin toxicity
Efavirenz	Increase voriconazole oral maintenance dose from 200 mg to 400 mg every 12 hours (100–200 mg every 12 hours if <40 kg) and reduce efavirenz dose by 50% to 300 mg/day
Rifabutin (according to FDA contraindicated as rifampicin)	According to EMA, increase oral voriconazole maintenance dose from 200 to 350 mg every 12 hours (100–200 mg every 12 hours if <40 kg) and intravenous maintenance dose to 5 mg/kg every 12 hours; monitor for rifabutin toxicity
Other drugs (apart from ritonavir, their levels are increased by voriconazole)	
Low dose ritonavir (100 mg every 12 hours)	Co-administration decreases levels of both voriconazole and ritonavir; better avoided
Cyclosporine, omeprazole, tacrolimus and warfarin	Their blood levels are increased by voriconazole and their dose should be reduced (by half for cyclosporine and by two-thirds for tacrolimus). Monitor serum levels of cyclosporine and tacrolimus or INR for warfarin
Other drugs such as benzodiazepines, opioid analgesics (for example, oxycodone or fentanyl), sulfonylureas, statins, vinca alkaloids, calcium channel blockers	Their levels are increased by voriconazole co-administration. Monitor closely for their side effects, discontinue if toxicity is suspected or consider decreasing dosage immediately when voriconazole is started

EMA, European Medicines Agency; FDA, Food and Drug Administration; INR, international normalized ratio.

As far as voriconazole dosing in special populations is concerned, supratherapeutic concentrations (4 mg/kg actual body weight) have recently been documented as a risk [86]. Therefore, dosing voriconazole based on an ideal body weight or adjusted body weight has been recommended for morbidly obese patients [86,87]. Conversely, clearance of voriconazole during continuous veno-venous hemofiltration (CVVH) was not clinically significant, so voriconazole dose adjustment in critically ill patients undergoing the standard method of CVVH is not required [88].

Several recent papers have underlined the crucial role of adequate plasma levels for maintaining efficacy during treatment of invasive fungal infections in immunocompromised patients [87,89-91]. A trough concentration of at least 1 mg/L was associated with an approximately 70% response rate in adult patients, and to date the recommended range is between 1 and 5.5 mg/L [70]. Interestingly, a reference laboratory experience of clinically achievable voriconazole bloodstream concentrations in a large number of subjects (n = 14,370) showed that 50.6% of samples were within the recommended trough range [77].

Although we still await definitive evidence-based guidelines on therapeutic drug monitoring of voriconazole, some practical indications, listed in order of importance, are summarized in Table 5.

**Table 5:** **Practical indications, listed in order of importance, when therapeutic drug monitoring of voriconazole might be useful**

**Clinical situation**	
●	Suspected treatment failure
●	Suspected suboptimal dosing - for example, due to interaction with other drugs such as phenytoin, in children or in cerebral infections Change in the administration of the drug from intravenous to oral route^a^
●	Suspected suboptimal absorption
●	Suspected non-compliance
●	Suspected neurologic toxicity possibly related to overdosing
●	Suspected other toxicity possibly related to overdosing

^a^As long as the patient is critical, intravenous therapy is preferred in order to avoid problems with absorption.

#### Pharmacological properties of echinocandins

The echinocandins are semisynthetic lipopeptides that act as noncompetitive inhibitors of 1,3-beta-D-glucan synthase, an enzyme complex within the fungal cell wall [92]. All the echinocandins exert *in vitro* and *in vivo* activity against *Aspergillus* spp. [93].

From a pharmacokinetic standpoint, the echinocandins are all similar for some aspects but differ for others [92]. All are highly bound to plasma protein, do not diffuse through the blood–brain barrier and/or the blood-ocular barrier, have a low propensity for drug-drug pharmacokinetic interaction (especially anidulafungin), are not renally cleared and have elimination half-lives long enough to allow once-daily administration. Recent studies suggest that the influence of continuous renal replacement therapy on anidulafungin, caspofungin or micafungin elimination in critically ill patients appears to be negligible, and that no dosage adjustments are needed for the echinocandins in patients undergoing CVVH ) [94-98].

It has been shown that hypoalbuminemic post-surgical patients might experience caspofungin underexposure due to increased clearance as a result of decreased plasma protein binding [99]. Likewise, a recent study in critically ill patients suggested that standard doses of anidulafungin resulted in lower exposure than in the general patient population, even if no correlation between anidulafungin exposure and plasma protein concentrations was established [99]. Additionally, it has been shown that dose optimization of caspofungin in obese patients may improve clinical success rates [100].

Although these issues are not expected to greatly affect echinocandin efficacy against *Candida* strains [101], they might become more relevant in the presence of less susceptible pathogens.

Although caspofungin is approved for second-line management of proven or probable IA at the standard dose of 50 mg once daily, it is worth noting that currently ongoing pharmacokinetic studies in patients with IA with higher doses ranging between 70 and 200 mg once daily suggest linear pharmacokinetics with no unpredictable accumulation across the investigated dosage range and good safety [102,103].

#### Pharmacological properties of liposomal amphotericin B

Amphotericin B is a polyene antibiotic that binds to the ergosterol present in the fungal membrane. Among the various lipidic formulations of amphotericin B, liposomal amphotericin B (LAmB) has the more favorable pharmacokinetic behavior in terms of achieving higher peak plasma levels, having lower intracellular penetration rates and lower clearance through the reticuloendothelial system [104]. Interestingly, both LAmB and amphotericin B lipid complex (ABLC) were shown to achieve therapeutically effective concentrations in the epithelial lining fluid of critically ill patients [105]. However, experimental animal models suggest that only LAmB may achieve adequate levels in the CSF [106] and the eye [107].

The pharmacokinetic-pharmacodynamic relationships of the two most widely used lipid formulations of amphotericin B (LAmB and ABLC) were shown to differ markedly in an *in vitro* lung model of IA, considering that the concentrations producing a 50% maximal effect were about four-fold lower for LAmB than for ABLC [108].

As far as LAmB dosing is concerned, it has been shown that dosages up to 10 mg/kg/daily gave no benefit for treatment of IA in comparison with the standard dose of 3 to 5 mg/kg/daily [57,109]. However it is worth noting that alternative dosing schedules based on higher dosages at longer dosing intervals are currently under evaluation for both prophylactic [110] and therapeutic [111] purposes.

Although potentially nephrotoxic, LAmB does not need dosage adjustment in the presence of renal insufficiency and recent clinical experience suggests that the impact of LAmB on the renal function of critically ill patients with impaired renal function was minimal [112,113].

### Outcome and prognostic factors

Only a few clinical studies have investigated the outcome of IA in critically ill patients. Different studies are difficult to compare due to the absence of specific clinical signs, different diagnostic criteria and different coexisting diseases recognized as risk factors [16].

Mortality rates for patients with proven or probable IA in the ICU range from 59% to 95% and seem to be higher in non-neutropenic patients [114]. A mortality rate of 60% was observed for immunocompromised patients compared to 89% in non-neutropenic patients (*P* = 0.007) [6]. In the latter group, fungal infection was proven to be the main cause of death for 8 patients (22.2%). Russo and colleagues [115] observed similar results: 14.3% of patients died as a direct consequence of *Aspergillus* infection. The mortality rate in these patients could be greater than in neutropenic patients. Compared to neutropenic patients, non-neutropenic patients could have a less symptomatic fungal infection with a complicated diagnosis, leading to suboptimal management and delayed therapy [6].

In a retrospective analysis of fungal infections in non-neutropenic patients, Garbino and colleagues [116] showed a mortality rate of 57.1% for patients with IA. Trof and colleagues [11] showed that IA diagnosis was established post-mortem in 38% of patients, 94% of whom did not receive antifungal treatment. These data could explain the results observed by Meersseman and colleagues [16] in a restrospective cohort study on 127 ICU patients with IA; patients with proven or probable infection without hematologic malignancy presented a two-fold increase in mortality rate compared with mortality expected by Simplified Acute Physiology Score II score.

Prognostic factors have been examined in a variety of studies. Isolation of *Aspergillus* in critically ill patients is associated with high mortality, irrespective of invasion or colonization [11]. Cornillet and colleagues [6] identified three factors associated with a poor prognosis: disseminated infection (100% mortality rate), co-infection (78% mortality rate) and bacterial pneumonia (78.5% mortality rate). In conclusion, it is possible that the overall mortality rate from IA is significantly higher in non-neutropenic patients.

## Conclusion

The management of IA in non-neutropenic, critically ill patients represents a challenge for clinicians. Features of IA in this cohort may contribute to a delay in diagnosis and, consequently, to commencement of adequate antifungal therapy. The complex underlying conditions and the non-specificity of symptoms in non-neutropenic patients may be confounding and lead to underdiagnosis and underestimates of the disease prevalence in this population. Furthermore, current guidelines are mainly designed for recognizing and managing IA in hematological patients with severe and prolonged neutropenia. Although recent advances in microbiological techniques (GM analysis, PCR, and so on) showed promising results in identifying IA also in non-conventional subsets of patients, such as the critically ill, a high level of suspicion of IA should be maintained especially when risk factors (for example, COPD, steroid use) are present. Voriconazole still represents the drug of choice for IA in non-neutropenic patients. Since mortality resulting from IA in non-neutropenic, critically ill patients appears to be higher than in immunocompromised patients and its management is problematic, studies on large cohorts and trials to better define the characteristics of IA are encouraged.

## Abbreviations

ABLC: Amphotericin B lipid complex
BAL: Bronchoalveolar lavage
COPD: Chronic obstructive pulmonary disease
CSF: Cerebrospinal fluid
CT: Computed tomography
CVVH: Continuous veno-venous hemofiltration
GM: Galatctomannan
IA: Invasive aspergillosis
LAmB: Liposomal amphotericin B
PCR: Polymerase chain reaction
